# Interstitial pneumonia with autoimmune features: an additional risk factor for ARDS?

**DOI:** 10.1186/s13613-017-0320-3

**Published:** 2017-09-18

**Authors:** Giacomo Grasselli, Beatrice Vergnano, Maria Rosa Pozzi, Vittoria Sala, Gabriele D’Andrea, Vittorio Scaravilli, Marco Mantero, Alberto Pesci, Antonio Pesenti

**Affiliations:** 1 Dipartimento di Anestesia, Rianimazione ed Emergenza-Urgenza, Fondazione IRCCS Ca’ Granda, Ospedale Maggiore Policlinico, Via Francesco Sforza 35, 20122 Milan, Italy; 2 Dipartimento di Emergenza-Urgenza, Ospedale San Gerardo, Monza, Italy; 3 Dipartimento di Medicina, Unità Operativa di Reumatologia, Ospedale San Gerardo, Monza, Italy; 4 Unità Operativa di Radiodiagnostica, Ospedale San Gerardo, Monza, Italy; 5 Dipartimento di Fisiopatologia Medico Chirurgica e dei Trapianti, Università degli Studi di Milano, Milan, Italy; 6 Dipartimento di Medicina e Chirurgia, Università Milano Bicocca, Monza, Italy; 7 Clinica Pneumologica, Ospedale San Gerardo, Monza, Italy

**Keywords:** Interstitial pneumonia with autoimmune features, Lung-dominant connective tissue disease, ARDS, ECMO

## Abstract

**Background:**

Interstitial pneumonia with autoimmune features (IPAF) identifies a recently recognized autoimmune syndrome characterized by interstitial lung disease and autoantibodies positivity, but absence of a specific connective tissue disease diagnosis or alternative etiology. We retrospectively reviewed the clinical presentation, diagnostic workup and management of seven critically ill patients who met diagnostic criteria for IPAF. We compared baseline characteristics and clinical outcome of IPAF patients with those of the population of ARDS patients admitted in the same period.

**Results:**

Seven consecutive patients with IPAF admitted to intensive care unit for acute respiratory distress syndrome (ARDS) were compared with 78 patients with ARDS secondary to a known risk factor and with eight ARDS patients without recognized risk factors. Five IPAF patients (71%) survived and were discharged alive from ICU: Their survival rate was equal to that of patients with a known risk factor (71%), while the subgroup of patients without risk factors had a markedly lower survival (38%). According to the Berlin definition criteria, ARDS was severe in four IPAF patients and moderate in the remaining three. All had multiple organ dysfunction at presentation. The most frequent autoantibody detected was anti-SSA/Ro52. All patients required prolonged mechanical ventilation (median duration 49 days, range 10–88); four received extracorporeal membrane oxygenation and one received low-flow extracorporeal CO_2_ removal. All patients received immunosuppressive therapy.

**Conclusions:**

This is the first description of a cohort of critical patients meeting the diagnostic criteria for IPAF presenting with ARDS. This diagnosis should be considered in any critically ill patient with interstitial lung disease of unknown origin. While management is challenging and level of support high, survival appears to be good and comparable to that of patients with ARDS associated with a known clinical insult

**Electronic supplementary material:**

The online version of this article (doi:10.1186/s13613-017-0320-3) contains supplementary material, which is available to authorized users.

## Background

The term “connective tissue disease” (CTD) refers to a heterogeneous group of disease that targets the connective tissues of the body. The autoimmune CTDs are caused by overactivity of the immune system, resulting in the production of autoantibodies. Their diagnosis is based on the combination of clinical history, physical examination and laboratory and radiologic findings, according to the established diagnostic criteria [1, 2].

Clinical presentation ranges from mild symptoms to life-threatening manifestations. Up to 30% of patients with CTD require intensive care unit (ICU) admission, and the CTD is frequently diagnosed during the ICU stay [3, 4]. The main reason for ICU admission is acute respiratory failure (ARF): type and frequency of lung involvement vary among the different CTDs [5], but a typical presentation is represented by interstitial lung disease (ILD) [6, 7].

The early recognition of the etiology of ILD (CTD, environmental exposures, drugs or idiopathic conditions) is crucial to choose the appropriate therapeutic strategy but can be really challenging. Nonetheless, a significant number of patients with ILD have clinical and/or serologic features suggesting an autoimmune etiology but do not fulfill established diagnostic criteria for a definite CTD: In these cases, different definitions have been proposed like undifferentiated CTD (UCTD) [8, 9], lung-dominant CTD (LD-CTD) [10, 11] and autoimmune-featured ILD (AIF-ILD) [12], but none of these is universally accepted. Recently, a consensus on the term “interstitial pneumonia with autoimmune features” (IPAF) has been reached by a joint European Respiratory Society (ERS)–American Thoracic Society (ATS) task force [13].

We present here for the first time a series of seven patients meeting the diagnostic criteria for IPAF requiring ICU admission for acute respiratory distress syndrome (ARDS) and multiple organ failure. Baseline characteristics and clinical outcome of IPAF patients were compared with those of the population of ARDS patients admitted in the same period.

## Methods

### Patient population

We retrospectively reviewed the electronic files of patients with a diagnosis of ARDS (according to the Berlin definition criteria) [14] admitted to the tertiary level ICUs of San Gerardo Hospital in Monza and of Fondazione IRCCS Ca’ Granda Ospedale Maggiore Policlinico in Milan (Italy) from May 2012 to October 2016. Based on the presence or absence of a recognized risk factor for ARDS among those listed in the Berlin definition criteria [14], patients were subdivided into two groups: (1) patients with a known risk factor; (2) patients without a known risk factor. The latter group was further subdivided into two subgroups according to the presence of diagnostic criteria for IPAF [13]: a. patients with IPAF (study group); b. patients without both a recognized risk factor and not fulfilling the criteria for IPAF.

The following data at ICU admission were recorded: demographics (sex, age), comorbidities, severity scores (SAPSII and SOFA), risk factors for ARDS, PaO_2_/FiO_2_ (P/F) ratio, intrapulmonary shunt (Qs/Qt), ventilator and respiratory mechanics parameters [positive end-expiratory pressure (PEEP), tidal volume (Vt), plateau pressure (*P*_plat_), driving pressure (Δ*P*), respiratory rate (RR), static respiratory system compliance (*C*_rs_)].

*P*_plat_ was measured during an end-inspiratory pause of at least 2 s ; Δ*P* was defined as *P*_plat_ − PEEP_tot_; static *C*_rs_ was calculated as Vt/Δ*P*.

The following outcome data were collected: use of prone positioning, need for extracorporeal membrane oxygenation (ECMO) support, duration of invasive mechanical ventilation (IMV) and ECMO (if applicable), ICU length of stay (ICU-LOS) and ICU mortality.

IPAF was diagnosed according to the criteria described by the joint ATS–ERS task force (Table S1) [13].

In all patients with IPAF (study group) and with ARDS without known risk factors, computed tomography (CT) of the chest and bronchoalveolar lavage (BAL) were performed within 2 days from ICU admission, and their findings were reviewed according to the ATS Guidelines [15, 16]. Timing, dose and schedule of administration of steroid therapy and other eventual immunosuppressive treatments were recorded. Episodes of ventilator-associated pneumonia (VAP) occurring in IPAF patients were analyzed. Finally, in patients with IPAF we obtained long-term follow-up data on lung morphology (by CT scan), respiratory function (by spirometry) and immunological status.

### Statistical analysis

Data are presented as absolute frequency (% of the included patients) or as median and interquartile range. To evaluate differences between patients’ groups, the Kruskal–Wallis test and Pearson’s Chi-squared test were utilized to compare continuous and nominal variables, respectively. Two-tailed values of *p* below 0.05 were considered statistically significant. The JMP 11 statistical program (SAS Institute Inc, Cary, NC) was used for statistical analysis.

## Results

**Fig. 1:**
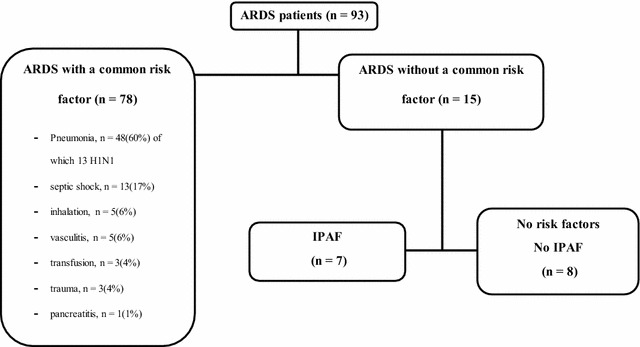
Patient population

Data from 93 patients with ARDS were reviewed. As previously described, patient population was subdivided into three groups (Fig. 1): (1) patients with a known risk factor for ARDS (*n* = 78); (2) patients without risk factors but fulfilling the diagnostic criteria for IPAF (*n* = 7); (3) patients without risk factors and not meeting the criteria for IPAF (*n* = 8).

Among the 78 patients with ARDS secondary to a known clinical insult, pneumonia was the most frequent risk factor (47 cases, 13 of viral origin), as shown in Fig. 1.

### Description of IPAF patients

Seven patients (four males and three females) with ARF due to a newly diagnosed ILD meeting the diagnostic criteria for IPAF were identified. Median age was 61 years (IQR 55–64); median SAPS II and SOFA scores at admission were 32 (32–36) and 11 (11–12.5), respectively. Five patients (71%) survived: Their median ICU stay was 54 (23–78) days and they were all discharged alive from the hospital. Two patients died 72 and 23 days after ICU admission, respectively (Table 1).

**Table 1:** Patient characteristics, ventilator and respiratory mechanics parameters (at admission) severity scores (at admission) and clinical outcomes of the seven IPAF patients included in the study

PT	Age (years)	Sex	PEEP (cmH_2_O)	*P*_plat_ (cmH_2_O)	Δ*P* (cmH_2_O)	*C*_rs_ (mL/cmH_2_O)	P/F (mmHg)	Qs/Qt	SAPS II score	SOFA score	ICU stay (days)	MV duration (days)	ECMO duration (days)	Outcome
PT 1	62	M	16	25	9	42	80	51	37	11	84	81	64	Alive
PT 2	45	M	12	30	18	22	97	54	37	10	72	72	63	Dead
PT 3	50	M	14	26	12	30	141	35	24	11	95	88	53	Alive
PT 4	66	F	10	31	21	12	120	NA	36	13	23	21	0	Alive
PT 5	61	M	14	29	15	26	163	NA	32	14	10	10	0	Alive
PT 6	74	F	12	26	14	27	77	25	32	12	23	23	0 (ECCO_2_R)	Dead
PT 7	61	F	12	24	14	14	122	26	32	11	54	49	17	Alive

*P*_plat_: end-inspiratory plateau pressure; Δ*P*: driving pressure; *C*_rs_: respiratory system compliance; P/F: PaO_2_/FiO_2_ ratio; Qs/Qt: intrapulmonary shunt; *SAPS* Simplified Acute Physiology Score, *SOFA* Sequential Organ Failure Assessment, *ICU* intensive care unit, *MV* mechanical ventilation, *ECMO* extracorporeal membrane oxygenation; ECCO_2_R: extracorporeal CO_2_ removal; *NA* not available

All patients were admitted to the hospital for acute dyspnoea and hypoxemia. Extrathoracic symptoms were few and nonspecific (fatigue, weight loss, fever), and none had skin lesions or arthritis. No patient had previous history of ILD and/or CTD, and detailed medication and occupational history were negative.

According to the oxygenation criteria established by the Berlin definition [14], three patients had moderate and four had severe acute respiratory distress syndrome (ARDS); however, in none of them a “known clinical insult” [14] could be identified as the cause of ARDS. At admission, median P/F ratio was 120 mmHg (range 80–163) and median respiratory system compliance 26 ml/cmH_2_O. All patients required intubation and invasive mechanical ventilation (MV). At first day of MV, median positive end-expiratory pressure (PEEP) was 12 cmH_2_O, plateau airway pressure 26 cmH_2_O and tidal volume 380 mL (Table 1). The time course of selected parameters during the ICU stay is depicted in Additional file 1: Figure S1. The following rescue therapies were applied: recruitment maneuvers (all patients), inhaled nitric oxide (three patients) and prone positioning (four patients). The median duration of MV was 49 days (21–81) and six subjects (85%) were tracheostomized. Five patients (71%) required an extracorporeal respiratory support with veno-venous extracorporeal membrane oxygenation (ECMO) in four cases and with low-flow extracorporeal CO_2_ removal (ECCO_2_R) with a dedicated device (Prolung™, Estor) in one case. Three of these patients survived and the median ECMO duration was 53 days (17–63).

All patients needed vasopressor support during the ICU stay and two presented with shock at admission. One patient required continuous renal replacement therapy.

Bacterial, viral or fungal infections were excluded in all patients by means of a complete microbiological workup, consisting of cultural, serological and molecular biology tests on blood, urine and BAL samples.

Other potentially reversible causes, such as pneumothorax, pulmonary embolism, left heart failure, acute eosinophilic pneumonia and hypersensitivity pneumonia, were ruled out.

Findings of BAL samples and CT scans are detailed in Table 2. Briefly, cytomorphological analysis of BAL showed mixed alveolitis with significant lymphocytosis (>30%) in three cases and significant neutrophilia (>50%) in two other patients, without signs of viral inclusion or diffuse alveolar hemorrhage.

**Table 2:** Autoantibodies pattern, BAL and CT scan findings of IPAF patients

PT	Autoantibodies	BAL	CT findings at presentation	ILD pattern at CT	CT findings at follow-up
PT 1	ANA; SSA/Ro52; anti-centromere	Lymphocytic cellular pattern (MA 42%; L 35%; PMN 14%; E 2%)	Extensive GGO with crazy-paving mainly in dependent zones and with subpleural sparing; consolidations in costophrenic sulci; some cystic lesions mainly in lower areas	LIP	Minimal diffuse GGO and subpleural reticulations; rare traction bronchiolectasis; enlarged cystic lesions (20 months)
PT 2	SSA/Ro52	Neutrophilic cellular pattern (MA 9%; L 0%; PMN 91%; E 0%); Foam cells +++	Focal subpleural and peribroncovascular consolidations in upper lobes and extensive consolidations of RLL, with air bronchogram	OP	Extensive parenchymal fibrosis with large bilateral pleural effusions (after 2 months of ICU stay)
PT3	SSA/Ro52	Physiological cellular pattern (MA 93%; L 2%; PMN 5%, E 0%); Foam cells +++	Subpleural consolidations with air bronchogram, mainly in lower lobes, with perilobular consolidations in RUL	OP	Diffuse reticulations with architectural distortion and subpleural curvilinear lines; limited traction bronchiectasis (17 months)
PT 4	ANA; SSA/Ro52	Neutrophilic cellular pattern (MA 9%; L 9%; PMN 83%; E 5%)	Limited gravity-dependent consolidations with air bronchogram and GGO; some areas of perilobular consolidations	OP	Gradual development of extensive GGO with reticulations, mainly in lower lobes with traction bronchiectasis (24 months)
PT 5	Anti-Jo1; SSA/Ro52	Lymphocytic cellular pattern (MA 27%; L 57%; PMN 14%; E 2%)	Diffuse GGO with subpleural sparing and crazy paving; subpleural consolidations mainly dorsal; initial signs of fibrosis with corkscrew-like traction bronchiectasis in RUL consolidated areas	AIP/DAD	Minimal subpleural GGO with reticulations; traction bronchiolectasis in RUL (23 months)
PT 6	ANA; SSA/Ro52	Lymphocytic cellular pattern (MA 60%; L 30%; PMN 10%; E 0%)	Bilateral consolidations with air bronchogram in lower lobes and dorsal segments of upper lobes. Some GGO in lower lobes and anterior segments of upper lobes. Limited reticulations anteriorly	OP	NA
PT 7	ANA (nucleolar pattern)	Physiological cellular pattern (MA 95%; L 1%; PMN 3%, E 1%)	Bilateral quite extensive GGO in upper and lower lobes, with subpleural and peribronchovascular distribution, associated with minimal reticulations in upper lobes and limited consolidations in lower lobes	OP/NSIP overlap	NA

*ANA* antinuclear antibodies, *RUL* right upper lobe, *GGO* ground-glass opacities, *RLL* right lower lobe, *MA* macrophages, *L* lymphocytes, *PMN* polymorphonuclear cells, *E* eosinophils, *LIP* lymphocytic interstitial pneumonia, *OP* organizing pneumonia, *AIP* acute interstitial pneumonia, *DAD* diffuse alveolar damage, *NA* not available

**Fig. 2:**
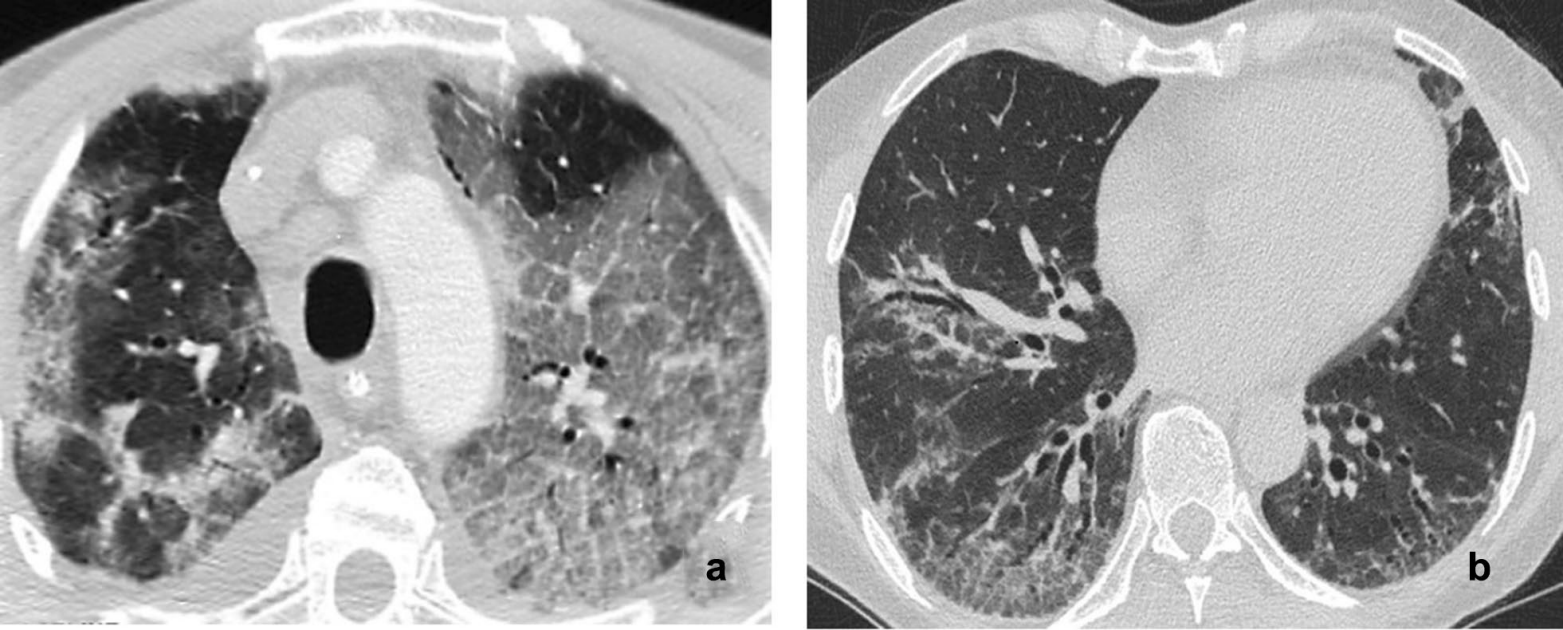
**a** Contrast-enhanced computed tomography scan of the thorax of Patient 5 at the level of the carina at ICU admission (slice thickness 2 mm). The picture shows bilateral, diffuse ground-glass opacities with partial subpleural sparing and crazy-paving pattern. **b** Follow-up high-resolution computed tomography scan of the thorax of Patient 4 at the level of the middle lobe (slice thickness 1 mm). The picture shows bilateral, mainly dorsal ground-glass opacities with reticulations and traction bronchiectasis

At CT scans, consolidations were present in all cases and mainly found in subpleural caudal areas, often associated with perilobular opacities (Fig. 2a; Additional file 1: Figure S2). Ground-glass opacities, in some cases with subpleural sparing, were also quite common but more diffuse and predominant at follow-up scans (Fig. 2b; Additional file 1: Figure S2). In four patients, mediastinal or hilar lymphadenopathies (with a diameter greater than 10 mm) were observed, while pleural effusion was present in only one case. Of the five patients who survived, two had limited or widespread organizing pneumonia (OP) consolidations; one had typical features of AIP/DAD with ground-glass opacities, patchy consolidations and fibrotic changes; one had extensive ground-glass opacities in lower lobes with some cysts suggestive of lymphocytic interstitial pneumonia (LIP) but with atypical extensive superimposition of “crazy-paving” pattern; the last one had and OP/nonspecific interstitial pneumonia (NSIP) overlap pattern. Both patients who died had an OP pattern at presentation, but in one of them the initial OP pattern rapidly progressed to acute interstitial pneumonia/diffuse alveolar damage (AIP/DAD) pattern and he subsequently developed striking extensive fibrotic parenchymal involvement. Follow-up scans were obtained in the first four surviving patients after a median interval from hospital discharge of 20 months. Patients with OP pattern at presentation developed ground-glass opacities both in new parenchymal territories and in previously consolidated areas. Three patients developed mild to moderate signs of fibrosis (traction bronchiectasis and mild parenchymal distortion), but no patient developed honeycombing. Representative images from baseline and follow-up CT scans of the first five patients are presented in Additional file 1: Figure S2.

A complete autoantibody panel was obtained, consisting of antinuclear antibodies (ANA), anti-SSA/Ro52, anti-SSB/LA, anti-ribonucleoprotein, anti-Scl70, anti-Smith, anti-Jo1, anti-centromere, anti-double-stranded DNA, anti-neutrophil cytoplasmic antibody (ANCA), cyclic anti-citrullinated peptide and rheumatoid factor (RF). All patients had anti-SSA/Ro52; one patient had high titer ANA and anti-centromere autoantibodies and one was positive for anti-Jo1. Since no other causes of ILD were identified, extrathoracic symptoms were nonspecific and pulmonary manifestations were by far the most important, all patients met the criteria for IPAF.

All patients received immunosuppressive therapy while in ICU, after a median interval of 10 days from admission. Five of them received high doses of methylprednisolone (1000 mg/day for 3 days and then 1 mg/kg/day), followed by cyclophosphamide (10–15 mg/kg every 2–3 weeks); they also received iv immunoglobulin infusion, and two were treated with cyclosporine for inadequate response to cyclophosphamide. The remaining two patients were treated with methylprednisolone (1 mg/kg/day) without loading dose.

Five patients (71%) developed a ventilator-associated pneumonia, after a median time interval of 19 (10–31) days from admission: Pneumonia was caused by multidrug-resistant Gram-negative bacteria in three cases and by Aspergillus fumigatus in one case.

The median follow-up of the first four surviving patients was 21 months. At the last visit, forced vital capacity range was 77–112% and diffusing lung capacity for carbon monoxide 39–95% of predicted and no patient requires oxygen supplementation. Immunological follow-up examinations showed that anti-SSA/Ro52 became negative in three out of four patients. Maintenance immunosuppressive therapy with low-dose prednisone, azathioprine and mycophenolate is ongoing in three patients.

All IPAF patients, except from the two who died, gave their written informed consent to publication. Due to the retrospective nature of the study and since written consent was obtained from the study patients, ethics committee approval was waived according to local regulations.

### Comparison between IPAF patients and control population

Table 3 shows the comparison of baseline characteristics and clinical outcome among the three patient groups. We did not find significant differences in regard to demographics, severity of hypoxemia (P/F ratio and Qs/Qt) and ventilator and respiratory mechanics parameters. IPAF patients tended to have a higher median SOFA score compared to both control groups (11 vs 8 vs 8.5; *p* = 0.068) and a higher frequency of use of prone positioning (86 vs 53 vs 43%; *p* = 0.197) and ECMO (71 vs 41 vs 25%; *p* = 0.176). ICU survival of IPAF patients was exactly equal to that of patients with a known risk factor (71%), while it was markedly higher than that of the patients without risk factors (38%); however, due to the small number of patients, this difference was not statistically significant. Compared to the other subgroups, IPAF patients had significantly longer median ICU-LOS (54 vs 19 vs 15.5 days; *p* = 0.0045) and duration of IMV (49 vs 17 vs 15.5 days; *p* = 0.031) and ECMO (53 vs 9.5 vs 28; *p* = 0.006).

**Table 3:** Patient characteristics, severity scores at admission, ventilator and respiratory mechanics parameters at admission and clinical outcome data in the three groups of patients included in the study

	ARDS with known risk factors	No risk factors	IPAF	*p*
Gender (male)	54 (69%)	1 (25%)	4 (57%)	0.763
Age (years)	55 (45–67.25)	58.5 (38.5–72.25)	61 (50–66)	0.726
SAPS II	41.5 (31–50.5)	42 (35–59)	32 (32–37)	0.133
SOFA	8 (5.75–12)	8.5 (8–11)	11 (11–13)	0.068
PaO_2_/FiO_2_ (mmHg)	93 (67.75–125.25)	103 (62.75–150.75)	120 (80–141)	0.515
PEEP (mmHg)	12.5 (10–15)	10 (7.25–14.75)	12 (10–14)	0.303
Compliance (mL/cmH_2_O)	29.4 (24–37)	26.5 (16.25–43.5)	26 (14–30)	0.336
Intrapulmonary shunt (%)	35 (25–46.5)	33 (14.5–40.5)	35 (25.5–52.5)	0.588
Plateau pressure (cmH_2_O)	28 (26–30)	27.5 (22.5–29.75)	26 (25–30)	0.796
Driving pressure (cmH_2_O)	14 (11–18)	15.5 (10.25–21.25)	14 (12–18)	0.977
ECMO	32 (41%)	2 (25%)	5 (71%)	0.176
Tracheostomy	45 (58%)	3 (43%)	5 (71%)	0.557
Pronation	41 (53%)	3 (43%)	6 (86%)	0.197
Survival	55 (71%)	3 (38%)	5 (71%)	0.159
ICU length of stay (days)	19 (8.75–32.25)*	15.5 (5.5–35.75)*	54 (23–84)	0.045
ECMO duration (days)	9.5 (6–13)*	28 (8–48)	53 (17–63)	0.006
IMV duration (days)	17 (6–28.5)*	15.5 (5.5–34.25)*	49 (21–81)	0.031

Data are presented as absolute frequency (% of the included patients) or as median and interquartile range

*IPAF* interstitial pneumonia with autoimmune features, *SAPS II* Simplified Acute Physiology Score, *SOFA* Sequential Organ Failure Assessment, *PEEP* positive end-expiratory pressure, *ECMO* extracorporeal membrane oxygenation, *ICU* intensive care unit, *IMV* invasive mechanical ventilation

* *p* < 0.05 versus IPAF group

In all the eight control patients without risk factors, microbiological and immunological screening resulted negative. Revision of chest CT scans and BAL performed in these patients did not allow the identification of a typical radiologic or cytomorphologic pattern, similarly to what observed IPAF patients. Of note, in all these patients a course of corticosteroid therapy was performed, mainly for “nonresolving ARDS.”

## Discussion

In this paper, we reviewed the clinical presentation, diagnostic workup and management of seven critically ill patients with IPAF. Our patients had an extremely severe clinical picture, with ARDS and multiple organ failure; they required prolonged mechanical ventilation and, in three cases, prolonged ECMO support. To the best of our knowledge, this is the first description of a cohort of patients with IPAF requiring ICU admission and invasive ventilation. Compared to the control population of ARDS patients admitted in the same period, ICU survival of patients with IPAF was equal to that of patients with ARDS associated with a known risk factor, while it was markedly higher than that of the subgroup of patients without risk factors (71 vs 38%). Hence, in our case series, IPAF patients had similar baseline characteristics and outcome to patients with ARDS associated with a recognized clinical insult, while patients without a known risk factor had a worse outcome. Of note, despite similar severity of hypoxemia and impairment of respiratory mechanics, IPAF patients had a significantly higher ICU-LOS and duration of IMV and required more frequently prone positioning and ECMO. Our findings confirm the data of Gibelin et al. [17] on a large series of ARDS patients: they observed that patients lacking exposure to common risk factors had a significantly higher mortality (66%) than other ARDS patients and found that the absence of known risk factors was independently associated with mortality (adjusted OR 2.06). Of note, De Prost et al. [18] recently published a secondary analysis on the cohort of ARDS patients without risk factors included in the LUNG SAFE study: ARDS without risk factors accounted for 8.3% of all ARDS cases and in 80% of these patients the etiology of ARDS was not identified.

Identification of the etiology of ILDs and their management remain a clinical challenge. The low incidence of ILD-associated ARF requiring ICU admission is a major obstacle to the assessment of outcome predictors and treatment optimization. Moreover, available studies report a high mortality among patients requiring invasive MV [7, 19, 20], ranging from 47 to 89.7%. For these reasons, ICU physicians tend to be reluctant to admit patients with ILD of uncertain etiology and are even more reluctant to administer immunosuppressive drugs in the absence of a definite diagnosis of an autoimmune disease.

Many authors have underlined the need to categorize patients with prevalent pulmonary involvement in probable autoimmune origin but with insufficient diagnostic criteria for a definite CTD. The rheumatologist would classify these patients as UCTD [8]; however, UCTD usually identifies patients with milder disease, nonspecific clinical features and low incidence of pulmonary involvement [2, 9].

Thus, Vij proposed the definition AIF-ILD [12]: Diagnostic criteria are the presence of ILD associated with at least one symptom/sign and one abnormal serologic test. In Vij’s retrospective review of 200 patients with ILD, 63 were classified as AIF-ILD and had significantly lower survival rates than patients with ILD associated with a definite CTD.

An alternative classification has been presented by Fischer, who proposed the definition of LD-CTD for “cases where ILD has a rheumatologic flavor as supported by specific autoantibodies and yet does not meet criteria for a defined CTD based on the lack of adequate extrathoracic features” [11]. However, ICU physicians are still not familiar with these diagnostic categories: Neither AIF-LD nor LD-CTD was considered in the large survey published by Dumas on 363 critically ill patients with systemic rheumatic diseases [21].

In the attempt to create consensus, an ERS-ATS task force recently proposed the definition of IPAF [13], based on the combination of at least one feature from at least two of three diagnostic domains: clinical (specific extrathoracic manifestations), serologic (specific circulating autoantibodies) and morphologic (suggestive radiologic or histopathologic pattern). The main characteristics of the classification are summarized in Additional file 1: Table S1.

All our patients but one were positive for anti-SSA/Ro52: Ro52 is a protein implicated in the process of ubiquitination and is upregulated at sites of autoimmune inflammation [22]. Several studies have demonstrated that anti-Ro52 is associated with ILD in patients with CTD, particularly in anti-synthetases syndromes [23]. Anti-tRNA synthetases were detected only in one of our patients, who had anti-Jo1 positivity but did not meet the diagnostic criteria for the anti-synthetase syndrome. However, diagnostic assays for anti-tRNA synthetases other than anti-Jo1 were not routinely available in our hospitals before 2015, and this might have reduced our ability to diagnose the disease.

As noted above, the definition of IPAF requires the presence of morphologic features, namely nonspecific interstitial pneumonia (NSIP), organizing pneumonia (OP) or lymphoid interstitial pneumonia (LIP) patterns at HRTC or lung biopsy [13]. Histologic and radiologic presentation in these patients is unspecific and variable, as recently demonstrated by Omote in a series of 44 patients with LD-CTD who underwent open lung biopsy [24]: the major histologic patterns were usual interstitial pneumonia (UIP) followed by NSIP, and UIP pattern was associated with worse prognosis. Similar findings were described in two recent studies on larger cohorts of patients meeting the diagnostic criteria for IPAF: More than half of the patients had a high-confidence diagnosis of UIP pattern on CT that was associated with worse prognosis particularly when associated with honeycombing or pulmonary artery enlargement [25–27]. We did not identify any specific radiologic (CT scan) or cytologic (BAL) findings in IPAF patients; a significant lymphocytosis in BAL, which strongly suggests an autoimmune etiology, was indeed observed only in three patients. Interestingly, none of these patients had a UIP pattern on CT scan, and this may explain the good clinical outcome of our cohort. Interestingly, lack of a typical CT scan and BAL findings was observed also in control patients without common risk factors. However, we acknowledge that the correlation between lung histology and radiologic pattern at CT scan may be quite poor, especially in critically ill patients undergoing mechanical ventilation.

One important limitation of our study is the lack of histologic data, due to the high risk of serious complications when a lung biopsy is performed during mechanical ventilation and ECMO. We acknowledge that lung histology is extremely helpful in patients with ARDS of unknown origin or in cases of nonresolving ARDS, since it can provide important diagnostic and prognostic information and guide patient management [28, 29]. Another limitation resides in the very small number of patients, which limits statistical power of the analysis even in case of large differences among the subgroups; moreover, the limited sample size precluded the possibility of performing a multivariate analysis of risk factors associated with mortality.

To the best of our knowledge, this is the first report of a series of critical patients fulfilling the diagnostic criteria for IPAF requiring ICU admission for ARDS. The number of patients is small, but the patient population is very well characterized and the clinical condition is rare. We think that our patient series demonstrates that the possibility of an autoimmune etiology and in particular the diagnosis of IPAF must be considered in any critically ill patient with “ARDS” according to the Berlin definition criteria but without a known risk factor. Once the diagnosis is established, these patients should receive a “full code” treatment, including ECMO if necessary, especially if the CT scan does not show a UIP pattern with associated signs of fibrosis or pulmonary hypertension. Management of IPAF patients is really challenging, but they can have a very good outcome if the appropriate therapy is instituted: immunosuppressive treatment can lead to a significant improvement in pulmonary manifestations and should be initiated as soon as an infectious cause has been excluded.

However, selecting the appropriate diagnostic and therapeutic strategy is extremely complex and requires a multidisciplinary approach: the intensivist should become part of a team together with the rheumatologist, the pulmonologist, the radiologist and the pathologist.

## Conclusions

 Interstitial pneumonia with autoimmune features (IPAF) is a rare autoimmune form of interstitial lung disease that can present acutely with ARDS and multiple organ failure, requiring ICU admission and advanced life support measures (included ECMO, if needed). This diagnosis should be considered in any patient presenting with interstitial pneumonia and ARDS lacking exposure to common risk factors for ARDS. In our small cohort of patients, the clinical response to immunosuppressive therapy was good, with a survival rate equal to that of patients with ARDS associated with a known clinical insult. Findings of the present study need to be confirmed prospectively on larger patient series.

## Additional file

**Additional file 1.** Electronic Supplementary Material

## Abbreviations

AIF-ILD: Autoimmune-featured interstitial lung disease
ANA: Antinuclear antibodies
ARDS: Acute respiratory distress syndrome
ARF: Acute respiratory Failure
ATS: American Thoracic Society
BAL: Bronchoalveolar lavage
CT: Computed tomography
CTD: Connective tissue disease
ECMO: Extracorporeal membrane oxygenation
ERS: European Respiratory Society
ICU: Intensive care unit
ILD: Interstitial lung disease
IPAF: Interstitial pneumonia with autoimmune features
LD-CTD: Lung-dominant connective tissue disease
LIP: Lymphocytic interstitial pneumonia
MV: Mechanical ventilation
NSIP: Nonspecific interstitial pneumonia
OP: Organizing pneumonia
RF: Rheumatoid factor
UCTD: Undifferentiated connective tissue disease
UIP: Usual interstitial pneumonia
