# Supplementary material for: Interstitial pneumonia with autoimmune features: an additional risk factor for ARDS?
Source: Ann Intensive Care. 2017 Sep 18;7:98. doi: 10.1186/s13613-017-0320-3 (PMC5603462; doi:10.1186/s13613-017-0320-3)
Supplement: Supplementary file 1 — Additional file 1. Electronic Supplementary Material [file 13613_2017_320_MOESM1_ESM.pdf]

SUPPLEMENTAL DIGITAL CONTENT

Manuscript title: Interstitial Pneumonia with Autoimmune Features: an additional risk factor for ARDS?

Authors: Giacomo Grasselli, MD; Beatrice Vergnano, MD; Maria Rosa Pozzi, MD; Vittoria Sala, MD; Gabriele D'Andrea, MD; Vittorio Scaravilli, MD; Marco Mantero, MD; Alberto Pesci, MD; Antonio Pesenti, MD

Table S1: diagnostic criteria for Interstitial Pneumonia with Autoimmune Features

Classification criteria for "interstitial pneumonia with autoimmune features"

1. Presence of an interstitial pneumonia (by HRCT or surgical lung biopsy) *and*,
2. Exclusion of alternative aetiologies *and*,
3. Does not meet criteria of a defined connective tissue disease *and*,
4. At least one feature from at least two of these domains:
    - A. Clinical domain
    - B. Serologic domain
    - C. Morphologic domain

**Clinical domain**	**Serologic domain**	**Morphologic domain**
• Distal digital fissuring (*i.e.* "mechanic hands")<br><br>• Distal digital tip ulceration<br><br>• Inflammatory arthritis *or* polyarticular morning joint stiffness ≥60 min<br><br>• Palmar telangiectasia<br><br>• Raynaud's phenomenon<br><br>• Unexplained digital oedema<br><br>• Unexplained fixed rash on the digital extensor surfaces (Gottron's sign)	1. ANA ≥1:320 titre, diffuse, speckled, homogeneous patterns *or* a. ANA nucleolar pattern (any titre) *or* b. ANA centromere pattern (any titre)<br><br>2. Rheumatoid factor ≥2× upper limit of normal<br><br>3. Anti-CCP<br><br>4. Anti-dsDNA<br><br>5. Anti-Ro (SS-A)<br><br>6. Anti-La (SS-B)<br><br>7. Anti-ribonucleoprotein<br><br>8. Anti-Smith	1. Suggestive radiology patterns by HRCT:<br>a. NSIP<br>b. OP<br>c. NSIP with OP overlap<br>d. LIP<br><br>2. Histopathology patterns or features by surgical lung biopsy:<br>a. NSIP<br>b. OP<br>c. NSIP with OP overlap<br>d. LIP<br>e. Interstitial lymphoid aggregates with germinal centres<br>f. Diffuse lymphoplasmacytic infiltration (with or without lymphoid follicles)<br><br>3. Multi-compartment involvement (in addition to interstitial pneumonia):<br>a. Unexplained pleural effusion or thickening<br>b. Unexplained pericardial effusion or thickening<br>c. Unexplained intrinsic airways disease (by PFT, imaging or pathology)<br>d. Unexplained pulmonary vasculopathy

	9. Anti-topoisomerase (Scl-70)<br><br>10. Anti-tRNA synthetase (*e.g.* Jo-1, PL-7, PL-12; others are: EJ, OJ, KS, Zo, tRS)<br><br>11. Anti-PM-Scl<br><br>12. Anti-MDA-5	

Reproduced with permission from ref. n. 13

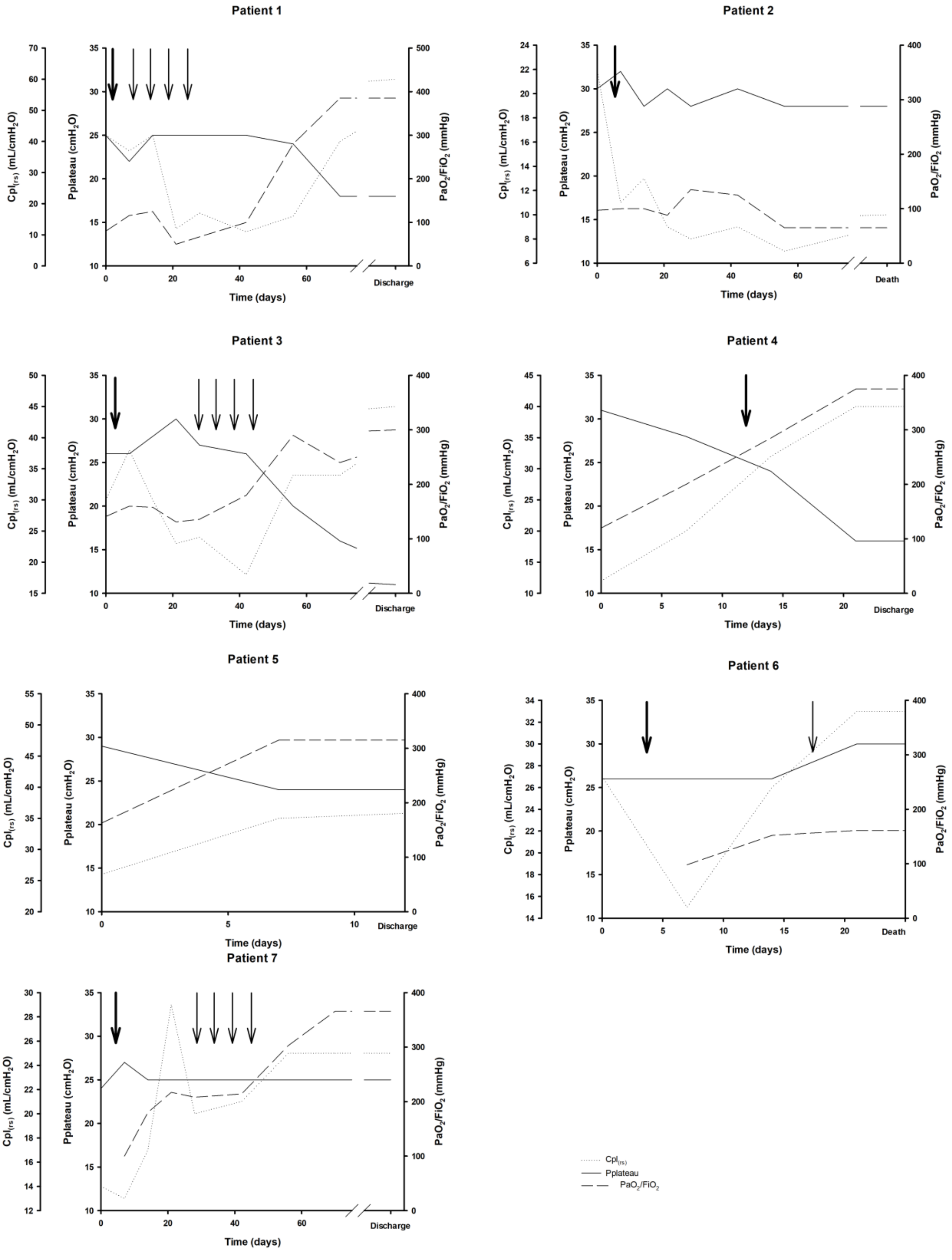

Figure S1: Time course of selected respiratory parameters during ICU stay of IPAF patients.

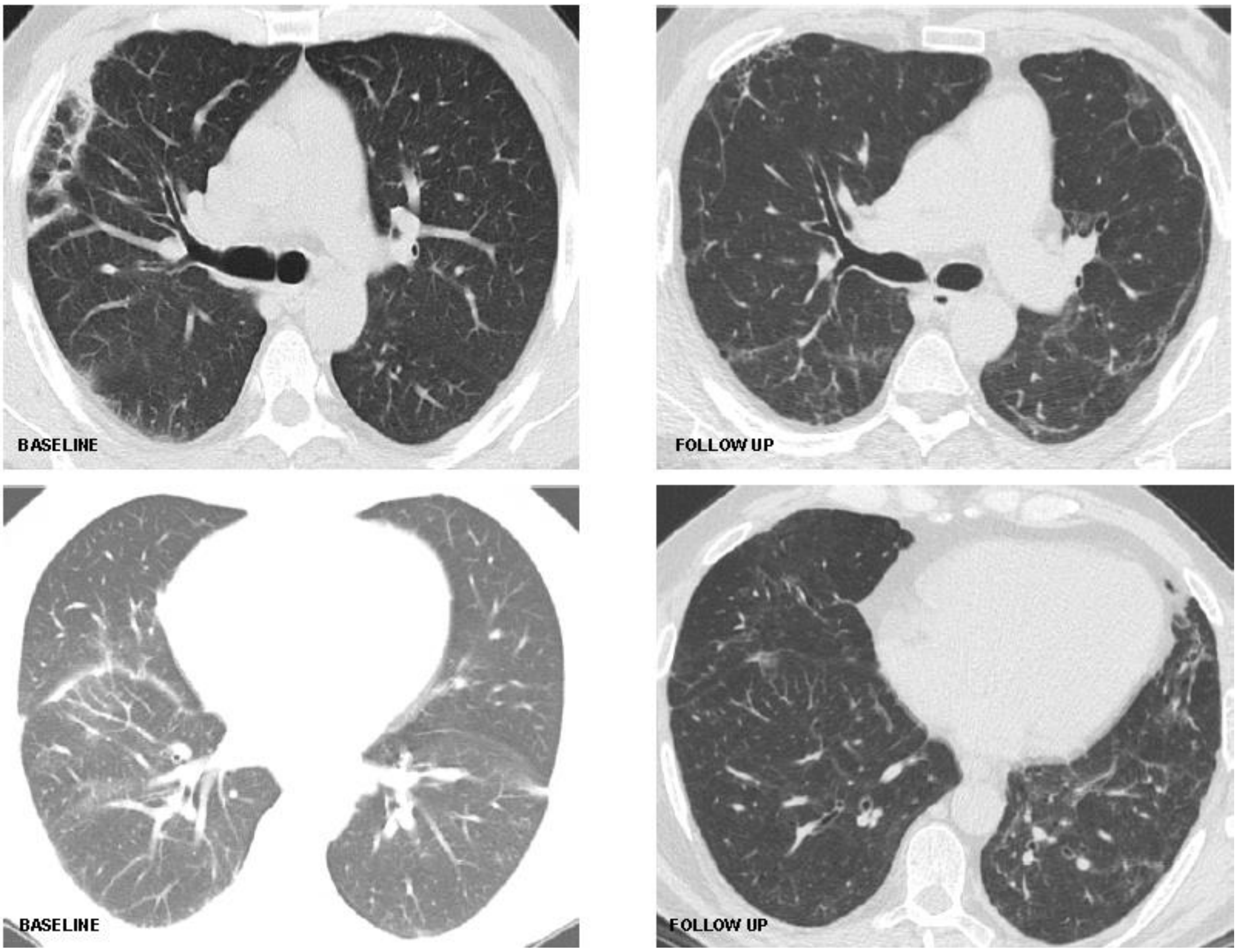

Fig S2. Patient 1. Baseline and follow-up images at the level of aortic arch (upper) and left atrium (lower). Initial CT findings include areas of ground glass opacities (GGO) with extensive crazy paving in the lower lobes associated with some cysts (LIP pattern). 20 months later follow up scan show minimal diffuse GGO and subpleural reticulations with rare traction bronchiolectasis and enlargement of some cysts.
